# lncRNA Oip5‐as1 attenuates myocardial ischaemia/reperfusion injury by sponging miR‐29a to activate the SIRT1/AMPK/PGC1α pathway

**DOI:** 10.1111/cpr.12818

**Published:** 2020-05-28

**Authors:** Xiaowei Niu, Shuangshuang Pu, Chun Ling, Jizhe Xu, Jing Wang, Shaobo Sun, Yali Yao, Zheng Zhang

**Affiliations:** ^1^ Heart Center The First Hospital of Lanzhou University Lanzhou Gansu China; ^2^ Gansu Clinical Medical Research Center for Cardiovascular Diseases The First Hospital of Lanzhou University Lanzhou Gansu China; ^3^ Gansu Key Laboratory of Cardiovascular Diseases The First Hospital of Lanzhou University Lanzhou Gansu China; ^4^ The Quality Improvement Project for the Diagnosis and Treatment of Complicated Cardiovascular and Cerebrovascular Diseases (2018) The First Hospital of Lanzhou University Lanzhou Gansu China; ^5^ The First School of Clinical Medicine Lanzhou University Lanzhou Gansu China; ^6^ The First People's Hospital of Chuzhou Chuzhou Anhui China; ^7^ Department of Gerontology The First Hospital of Lanzhou University Lanzhou Gansu China; ^8^ The College of Integrated Traditional Chinese and Western Medicine Gansu University of Chinese Medicine Lanzhou Gansu China

**Keywords:** apoptosis, microRNA‐29a, mitochondria, myocardial ischaemia/reperfusion injury, Opa‐interacting protein 5‐antisense transcript 1, sirtuin 1

## Abstract

**Objectives:**

Myocardial ischaemia/reperfusion (MI/R) injury is associated with adverse cardiovascular outcomes after acute myocardial infarction. However, the molecular mechanisms underlying MI/R injury are unclear. This study investigated the role of long non‐coding RNA (lncRNA) Oip5‐as1 in regulating mitochondria‐mediated apoptosis during MI/R injury.

**Materials and methods:**

Sprague‐Dawley rats were subjected to MI/R induced by ligation of the left anterior descending coronary artery followed by reperfusion. H9c2 cells were incubated under oxygen‐glucose deprivation/reoxygenation (OGD/R) conditions to mimic *in vivo* MI/R. RT‐qPCR and Western blot were used to evaluate gene and protein levels. CCK‐8 assay, biochemical assay and flow cytometric analysis were performed to assess the function of Oip5‐as1. The dual‐luciferase gene reporter assay and RIP assay were conducted as needed.

**Results:**

Oip5‐as1 expression was downregulated in the hearts of rats with MI/R and in H9c2 cells treated with OGD/R. Oip5‐as1 overexpression alleviated reactive oxygen species‐driven mitochondrial injury and consequently decreased apoptosis in MI/R rats and H9c2 cells exposed to OGD/R. Mechanistically, Oip5‐as1 acted as a competing endogenous RNA of miR‐29a and thus decreased its expression. Inhibition of miR‐29a reduced the oxidative stress and cytotoxicity induced by OGD/R. Overexpression of miR‐29a reversed the anti‐apoptotic effect of Oip5‐as1 in H9c2 cells treated with OGD/R. Further experiments identified SIRT1 as a downstream target of miR‐29a. Oip5‐as1 upregulated SIRT1 expression and activated the AMPK/PGC1α pathway by targeting miR‐29a, thus reducing the apoptosis triggered by OGD/R. However, these effects were reversed by a selective SIRT1 inhibitor, EX527.

**Conclusions:**

Oip5‐as1 suppresses miR‐29a leading to activation of the SIRT1/AMPK/PGC1α pathway, which attenuates mitochondria‐mediated apoptosis during MI/R injury. Our findings thus provide new insights into the molecular mechanisms of MI/R injury.

## 1 INTRODUCTION

Acute myocardial infarction (AMI) causes high morbidity and mortality worldwide.^1^ Currently, myocardial reperfusion by thrombolytic therapy or percutaneous coronary intervention is the most effective approach for AMI treatment.^2^ However, reperfusion itself can cause additional damage, a process known as myocardial ischaemia/reperfusion (MI/R) injury.^2^ MI/R injury contributes to the final myocardial infarct size, a major prognostic factor in patients with AMI.^2^, ^3^ Clinical treatment of MI/R injury is challenging, partly because of an incomplete understanding of the mechanisms underlying MI/R injury.^2^ Cardiomyocyte necrosis and apoptosis are suggested to play a key role in the progression of MI/R injury.^4^, ^5^, ^6^, ^7^ Multiple pathophysiological factors such as calcium overload, inflammation, oxidative stress and mitochondrial dysfunction occur after MI/R, thus inducing cardiomyocyte apoptosis.^2^, ^7^ Oxidative stress results from excessive reactive oxygen species (ROS) mainly from disruption of the mitochondrial respiratory chain during MI/R.^6^ Generation of ROS in turn interferes with oxidative phosphorylation and exacerbates mitochondrial dysfunction, thus creating a self‐amplifying vicious cycle.^5^ As such, preservation of mitochondrial function is considered an important therapeutic target for modulating cardiomyocyte apoptosis during MI/R injury.^2^, ^5^, ^7^

Recently, long non‐coding RNAs (lncRNAs) have emerged as new gene expression regulation and coordination factors.^8^ lncRNAs either downregulate or induce overexpression of protein‐coding genes by acting as epigenetic regulators, molecular scaffolds, molecular signals or decoys.^8^ One of their modes of action is interaction with microRNAs (miRNAs) in a mechanism known as competing endogenous RNAs (ceRNAs).^9^ lncRNAs act as ceRNAs by sponging miRNAs through sequence complementary to release mRNAs targeted by miRNAs.^9^ lncRNAs and miRNAs have been associated with a wide range of biological, physiological and pathological processes, such as in cardiovascular diseases.^10^, ^11^

OIP5‐AS1 (Opa‐interacting protein 5‐antisense transcript 1) was first identified as the mammalian homolog of the zebrafish transcript *cyrano* during analyses of the zebrafish and human transcriptomes.^12^ OIP5‐AS1 is an evolutionarily conserved lncRNA and is predominantly expressed in the cytoplasm.^12^, ^13^ OIP5‐AS1 is reported to be a key regulator in tumour growth and progression.^14^, ^15^, ^16^, ^17^, ^18^ For example, OIP5‐AS1 downregulation inhibits breast cancer progression by targeting SOX2 (sex‐determining region Y‐box 2) via miR‐129‐5p upregulation.^14^ OIP5‐AS1 promotes glioma oncogenesis by sponging miR‐367‐3p to modulate the expression of CEBPA (CCAAT/enhancer binding protein alpha).^16^ However, the role of OIP5‐AS1 in myocardial apoptosis following MI/R injury is unknown. Our pilot analysis showed that both Oip5‐as1 and miR‐29a are deregulated in a rat model with MI/R injury. Oip5‐as1 was found to contain a conserved miR‐29a binding site. Furthermore, a miR‐29a binding site was found at the 3′‐untranslated region (3′‐UTR) of SIRT1 (sirtuin 1) mRNA, which plays a key role in regulating the cardiomyocyte apoptosis induced by MI/R.^19^, ^20^

This study was therefore conducted to investigate the role of Oip5‐as1 in apoptosis triggered by mitochondrial dysfunction post‐MI/R injury. We showed for the first time that Oip5‐as1 sponged miR‐29a to upregulate the expression of SIRT1, which then activated the AMPK/PGC1α (AMP‐activated protein kinase/peroxisome proliferator‐activated receptor γ coactivator 1 alpha) signalling pathway to attenuate MI/R injury.

## 2 MATERIALS AND METHODS

### 2.1 Isolation of neonatal rat ventricular myocytes (NRVMs)

NRVMs were isolated from Sprague‐Dawley rats (1‐ to 3‐day‐old) purchased from the Experimental Animal Center of Lanzhou University (Lanzhou, Gansu, China). Ventricular myocardium was isolated and cut into l.0 mm pieces that were then digested in phosphate‐buffered saline (PBS) containing 0.04% collagenase type II and 0.07% trypsin (Sigma‐Aldrich). The cell suspension was filtered with a 200‐mesh sterile strainer and centrifuged at 1500 rpm for 5 minutes. The collected cells were then maintained in Dulbecco's modified Eagle medium (DMEM)/F12 (Gibco) supplemented with 10% foetal bovine serum (Gibco), 100 units/mL penicillin and 100 µg/mL streptomycin (Biological Industries) for 90 minutes. After the fibroblast adherence procedure, non‐adherent cells in the supernatant were transferred to cell culture dishes at a density of 1 × 10^6^ cells/mL. Then, 100 μmol/L 5‐bromodeoxyuridine (Selleckchem) was added to the culture medium for 48 hours to prevent the proliferation of non‐myocytes.^21^, ^22^ The study was approved by the Animal Care Committee of the First Hospital of Lanzhou University.

### 2.2 Cell line

The rat embryonic ventricular myocardial cell line, H9c2, was purchased from the China Infrastructure of Cell Line Resource (Beijing, China). H9c2 cells were cultured in DMEM (Gibco) in a humidified incubator containing 5% CO_2_ at 37°C.

### 2.3 Oxygen‐glucose deprivation/reoxygenation (OGD/R) injury

To establish an *in vitro* model of MI/R injury,^22^ NRVMs and H9c2 cells were washed thrice with PBS and cultured in serum and glucose‐free DMEM (Gibco). The cells were then incubated in a HeraCell VIOS 160i incubator (Thermo Fisher Scientific) flushed with a gas mixture containing 1% O_2_, 5% CO_2_ and 94% N_2_ for 3 hours. After OGD, the cells were incubated in complete culture medium under 5% CO_2_ and 95% air for 6 hours. Control cells were cultured in DMEM under normoxia.

### 2.4 CRISPR/dCas9 SAM system and cell transfection

Oip5‐as1‐overexpressing H9c2 cells were generated using the clustered regularly interspaced short palindromic repeats/deactivated Cas9 (CRISPR/dCas9) Synergistic Activation Mediator (SAM) system.^23^ The SAM complex containing lenti‐dCAS9‐VP64‐Puro and lenti‐sgRNA‐MS2‐P65‐HSF1‐Neo was purchased from Genechem. H9c2 cells were seeded in 6‐well plates and grown overnight. H9c2 cells were then transfected at a multiplicity of infection (MOI) of 20 for each virus. The culture medium was then changed after 12 hours. Puromycin (Genechem) and G418 (Genechem) were used to select the H9c2 cells with stable overexpression of Oip5‐as1 (oe‐Oip5‐as1) and the negative control (oe‐NC). The levels of Oip5‐as1 expression were determined by real‐time quantitative polymerase chain reaction (RT‐qPCR).

### 2.5 Transfection of cells with siRNAs

Small interfering RNAs (siRNAs) were used to silence the Oip5‐as1 gene. The siRNAs targeting Oip5‐as1 (si‐Oip5‐as1) and negative control siRNAs (si‐NC) were designed and synthesized by GenePharma. Lipofectamine 3000 reagent (Invitrogen) was used to transfect siRNAs into H9c2 cells following the manufacturer's instructions. After transfection, the original medium was replaced with fresh medium and H9c2 cells were incubated for 48 hours for further analysis. Target sequences for the Oip5‐as1 gene are listed in Supplementary Table S1.

### 2.6 Transfection of cells with miRNAs

Mimics and inhibitors of miR‐29a were synthesized by GenePharma. Scrambled RNAs were used as negative controls for miR‐29a mimics (mimic‐NC) and miR‐29a inhibitors (inhibitor‐NC). H9c2 cells were transfected with miR‐29a using the Lipofectamine 3000 reagent following the manufacturer's instructions. H9c2 cells were collected for analysis after 48 hours of transfection.

### 2.7 Cell treatment

H9c2 cells were exposed to 10 μmol/L of a selective SIRT1 inhibitor EX527 (Selleckchem) for 6 hours to explore the relationship between Oip5‐as1 and SIRT1/AMPK/PGC1α signal pathway in alleviating OGD/R‐induced injury.^24^

### 2.8 Cell viability assay

The cell counting kit‐8 (CCK‐8) assay was used to determine cell viability. H9c2 cells were first cultured in 96‐well plates at a density of 1 × 10^4^ cells/well. After respective treatments, 10 μL of CCK‐8 reagent (Bioss Biotech) was added to each well containing H9c2 cells. The plates were then incubated at 37°C for 2 hours in the dark. The optical density (OD) of each well was measured using an infinite M200 PRO microplate reader (Tecan) at a wavelength of 450 nm.

### 2.9 Determination of LDH

Lactate dehydrogenase (LDH) activity of H9c2 cells was measured using the LDH detection kit (Jiancheng) following the manufacturer's protocol. The OD of each well was measured at a wavelength of 450 nm.

### 2.10 Assessment of cell apoptosis

Apoptosis of H9c2 cells was analysed by flow cytometry using the Annexin V‐FITC/PI Apoptosis Detection Kit (Yeasen). H9c2 cells were washed twice with cold PBS and then suspended in 100 μL of binding buffer containing 5 μL Annexin V‐FITC and 10 μL PI in the dark. After 15 minutes of incubation, the cell suspensions were analysed using the NovoCyte flow cytometer (ACEA Biosciences). The percentage of apoptotic cells was calculated as the ratio of the Annexin V‐positive cells to the total cell population multiplied by 100%.

### 2.11 Measurement of intracellular ROS production

H9c2 cells were harvested and incubated in 10 μmol/L DCFH‐DA (Beyotime) at 37°C in the dark for 30 minutes. After incubation, the cells were washed thrice with PBS and their fluorescence was measured using the NovoCyte flow cytometer.

### 2.12 Biochemical assessment

H9c2 cells and heart tissues were used to evaluate oxidative stress parameters. As a specific indicator of ROS‐induced lipid peroxidation,^25^ 15‐F2t‐isoprostane levels were measured using a commercially available enzyme‐linked immunosorbent assay kit (Cayman Chemical). The activities of antioxidant enzymes, superoxide dismutase (SOD) and glutathione peroxidase (GPx), were determined using respective commercial kits (Beyotime). All procedures were performed according to the manufacturer's instructions.

### 2.13 Detection of the mitochondrial membrane potential

The mitochondrial membrane potential (MMP) was measured using the Mitochondrial Membrane Potential Assay Kit with JC‐1 (Beyotime) to determine mitochondrial membrane integrity. H9c2 cells were incubated in 1 mL of JC‐1 dye for 20 minutes at 37°C. Green (JC‐1 monomeric form) and red (JC‐1 aggregate form) fluorescences were easured using an Olympus IX71 microscope.

### 2.14 Dual‐luciferase gene reporter assay

Oip5‐as1 and the 3′‐UTR of Sirt1 for miR‐29a binding were amplified by PCR. The PCR products were then cloned downstream of the firefly luciferase gene in the pmirGLO Dual‐Luciferase miRNA Target Expression Vector (Promega), named Oip5‐as1‐WT and Sirt1‐WT, respectively. The Oip5‐as1 and Sirt1 3′‐UTR mutant plasmids were mutated in the putative miR‐29a binding sites and were named Oip5‐as1‐Mut and Sirt1‐Mut, respectively. HEK293T cells were cultured in 24‐well plates and transfected with the corresponding plasmids, miR‐29a mimics and NC mimics using the Lipofectamine 3000 reagent. After 48 hours, firefly and renilla luciferase activities in the lysates of transfected cells were measured on the GloMax 20/20 Luminometer (Promega) using the Dual‐Luciferase Reporter Assay System.

### 2.15 RNA immunoprecipitation assay

The RNA immunoprecipitation (RIP) assay was performed following the guidelines in the Magna RIP RNA‐Binding Protein Immunoprecipitation Kit (Millipore). H9c2 cells were harvested and lysed in RIP lysis buffer containing a protease inhibitor cocktail and RNase inhibitor. The cells lysates were then incubated with magnetic beads conjugated to anti‐Ago2 antibody or the negative control IgG (Abcam). The protein‐RNA complexes were then digested with Proteinase K buffer, and the co‐immunoprecipitated RNA was then isolated for RT‐qPCR analysis.

### 2.16 Rat model of MI/R injury

Sprague‐Dawley male rats weighing 250 ± 20 g were purchased from the Experimental Animal Center of Lanzhou University. The animals were housed under standard laboratory conditions at a temperature of 22 ± 2°C in a 12 hours light/dark cycle with free access to water and food. The study was conducted in accordance with the Guide for the Care and Use of Laboratory Animals published by the National Research Council. The study was approved by the Animal Care Committee of the First Hospital of Lanzhou University.

Rats were anesthetized with pentobarbital sodium (50 mg/kg ip) and ventilated using a small animal ventilator (Chengdu Technology Market). The chest was opened at the fourth intercostal space to expose the heart. The left anterior descending coronary artery was ligated using a 6‐0 Prolene suture at a point just below the left atrial appendage for 30 minutes and was then released for reperfusion. Sham‐operated rats received the same treatment without ligation.

### 2.17 *In vivo* gene therapy

Recombinant adeno‐associated virus (serotype 9) vectors with a cTNT promoter carrying Oip5‐as1 (AAV9‐Oip5‐as1) or empty AAV vectors (AAV9‐NC) were provided by Hanbio Biotechnology Co., Ltd.^26^ The rats underwent gene transfer by intramyocardial injection with 1 × 10^12^ vg/mL AAV9‐Oip5‐as1 or AAV9‐NC at 5 different sites (basal anterior, mid‐anterior, mid‐lateral, apical anterior and apical lateral) using a microsyringe (Hamilton). RT‐qPCR was performed to verify the efficiency of myocardial Oip5‐as1 overexpression.

### 2.18 Histological analysis

The cardiac infarct size was determined by 2,3,5‐triphenyltetrazolium chloride (TTC) staining (Solarbio) as described previously.^27^

Terminal deoxynucleotidyl transferase‐mediated dUTP nick end labelling (TUNEL) staining was used to detect apoptosis in heart tissues. The procedure was conducted using the One Step TUNEL Apoptosis Assay Kit (Beyotime) according to the manufacturer's protocol. The percentage of apoptotic cells (TUNEL‐positive nuclei/total nuclei) was calculated from randomly chosen fields per slide and was averaged for statistical analysis.

### 2.19 RT‐qPCR

Total RNA in cells and tissues was extracted using the TRIzol reagent (Invitrogen) following the manufacturer's instructions. PrimeScript RT reagent Kit with gDNA Eraser and TB Green Premix Ex Taq II (TaKaRa) were used to determine the expression of Oip5‐as1 and Sirt1. The Mir‐X miRNA First‐Strand Synthesis Kit and Mir‐X miRNA qRT‐PCR TB Green Kit (TaKaRa) were used to determine the expression of miR‐29a. β‐actin was used as an internal control in the determination of Oip5‐as1 and Sirt1 expression, whereas U6 was used in the case of miR‐29a expression. RT‐qPCR was run as per the manufacturer's instructions on a QuantStudio 5 Real‐Time PCR System (Thermo Fisher Scientific). The relative quantitative expression was determined using the 2^‐∆∆CT^ method.^28^ The primers (Supplementary Table S2) were synthesized by Sangon Biotech.

### 2.20 Detection of the Cyt‐c protein release

We isolated cytoplasmic proteins by removing the mitochondria using the Mitochondrial Extraction Kit (Solarbio) to analyse cytochrome c (Cyt‐c) release from the mitochondria into the cytoplasm. All procedures were performed according to the manufacturer's instructions. The expression of Cyt‐c protein in the cytoplasm was detected by Western blot analysis.

### 2.21 Western blot

Total proteins were isolated from cells and tissues lysed in RIPA buffer containing 1% PMSF and 1% phosphatase inhibitor cocktail (Solarbio). Protein concentrations were determined using the BCA Protein Assay Kit (Solarbio). Proteins were separated by SDS‐PAGE (10%‐15%), subsequently transferred onto a polyvinylidene fluoride membrane (Millipore) and blocked using 5% non‐fat milk or bovine serum albumin for 2 hours. The blocked membranes were incubated overnight with primary antibodies (Supplementary Table S3) at 4°C. The membranes then were incubated with secondary anti‐rabbit or anti‐mouse antibodies (ZSGB‐BIO) at room temperature for 2 hours. The protein bands in the membranes were detected using the ECL Kit (Millipore). Gel images were captured using Universal Hood II (Bio‐Rad) and were quantified using the ImageJ 1.51k software (National Institutes of Health).

### 2.22 Statistical analysis

All data are represented as means ± SD and were analysed with a two‐tailed Student's *t* test or one‐way ANOVA followed by a Student‐Newman‐Keuls (SNK) test using SPSS 25.0 software (SPSS). Graphs were drawn using GraphPad Prism 7.00 software (GraphPad Software). A two‐sided *P* value < .05 was considered statistically significant.

## 3 RESULTS

### 3.1 Oip5‐as1 is involved in the response to OGD/R‐induced injury

**FIGURE 1:**
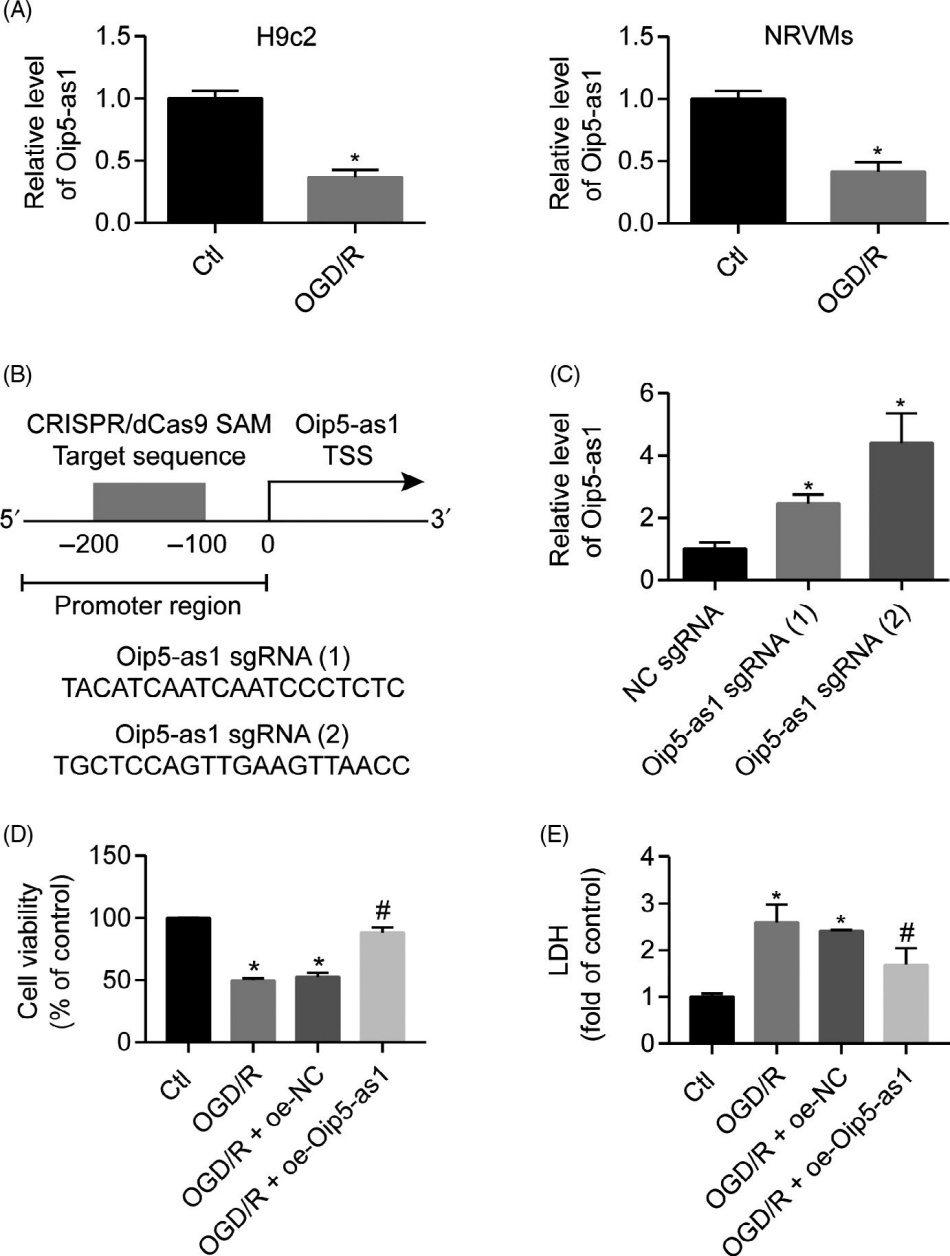
Oip5‐as1 is involved in the response to OGD/R‐induced injury. A, Oip5‐as1 expression levels in H9c2 cells and neonatal rat ventricular myocytes (NRVMs) after treatment with OGD/R. B, A schematic illustration of two sgRNA sequences and the CRISPR/dCas9‐SAM target site (grey) in the promoter region located −200 bp to −100 bp from the transcription start site (TSS) of the Oip5‐as1 gene. C, RT‐qPCR analysis of Oip5‐as1 expression in H9c2 cells transfected with the CRISPR/dCas9 SAM system. Oip5‐as1 sgRNA (2) was used in all experiments involving H9c2 cells. Cell viability (D) and LDH activity (E) of H9c2 cells transfected with Oip5‐as1 overexpressing vector (oe‐Oip5‐as1) or negative control vector (oe‐NC) under OGD/R conditions. Data are expressed as mean ± SD (n = 3). *P < .05 vs the control group. ^#^ *P* < .05 vs the OGD/R + oe‐NC group

To determine whether Oip5‐as1 was involved in MI/R injury, the expression levels of Oip5‐as1 in NRVMs and H9c2 cells were determined using RT‐qPCR. The results revealed that Oip5‐as1 was significantly downregulated in NRVMs and H9c2 cells subjected to OGD/R (Figure 1A).

Oip5‐as1‐overexpressing H9c2 cells were then generated using the CRISPR/dCas9 SAM system to help understand the Oip5‐as1 functions. Two approaches were designed for Oip5‐as1 upregulation mediated by the CRISPR/Cas9 system (Figure 1B). RT‐qPCR analysis revealed that Oip5‐as1 sgRNA (2) showed the best activation effect and thus was selected for subsequent experiments (Figure 1C). Oip5‐as1 overexpression increased cell viability (Figure 1D) but decreased LDH activity (Figure 1E) in OGD/R‐treated H9c2 cells. The oe‐NC showed no changes in the Oip5‐as1 expression, and H9c2 cell injury induced by OGD/R.

### 3.2 Oip5‐as1 alleviates H9c2 cell apoptosis induced by OGD/R

**FIGURE 2:**
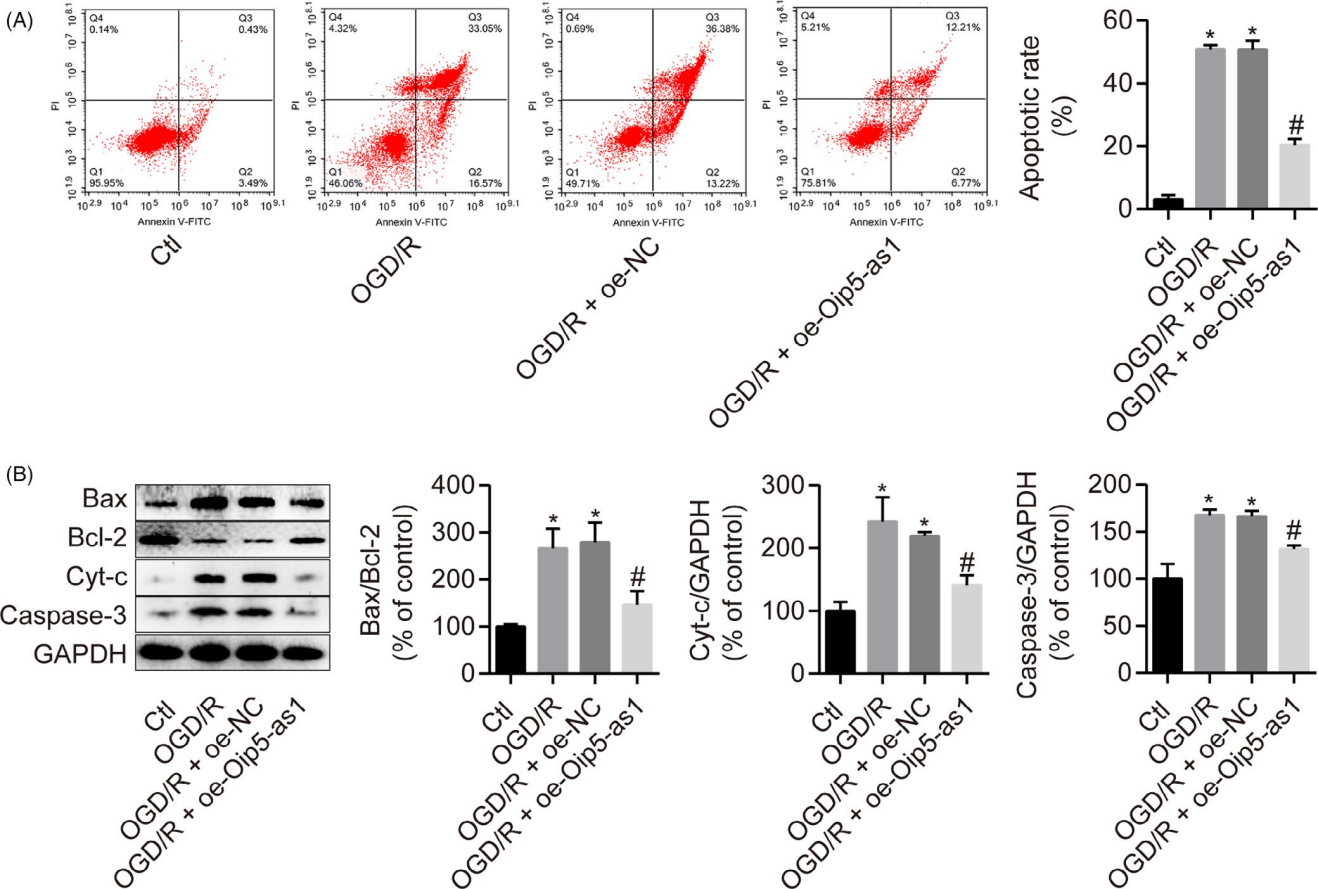
Oip5‐as1 alleviates H9c2 cell apoptosis induced by OGD/R. A, Cell apoptosis assessment by Annexin V/PI and flow cytometry in H9c2 cells treated with the Oip5‐as1 overexpression vector (oe‐Oip5‐as1) and OGD/R. B, Western blot and densitometric analysis of apoptosis‐related proteins (Bax, Bcl‐2, Cyt‐c and Caspase‐3) in H9c2 cells treated with oe‐Oip5‐as1 and OGD/R. Data are expressed as mean ± SD (n = 3). *P < .05 vs the control group. ^#^ *P* < .05 vs the OGD/R + oe‐NC group

The effect of Oip5‐as1 on OGD/R‐induced apoptosis in H9c2 cells was further investigated by flow cytometry and Western blot analysis. Overexpression of Oip5‐as1 significantly decreased the percentage of Annexin V‐positive cells induced by OGD/R (Figure 2A). Moreover, induced Oip5‐as1 expression suppressed the expression of apoptosis‐related proteins including Bax/Bcl‐2 ratio, Caspase‐3 and Cyt‐c in OGD/R‐treated H9c2 cells (Figure 2B).

### 3.3 Oip5‐as1 attenuates OGD/R‐induced oxidative stress and mitochondrial membrane depolarization in H9c2 cells

**FIGURE 3:**
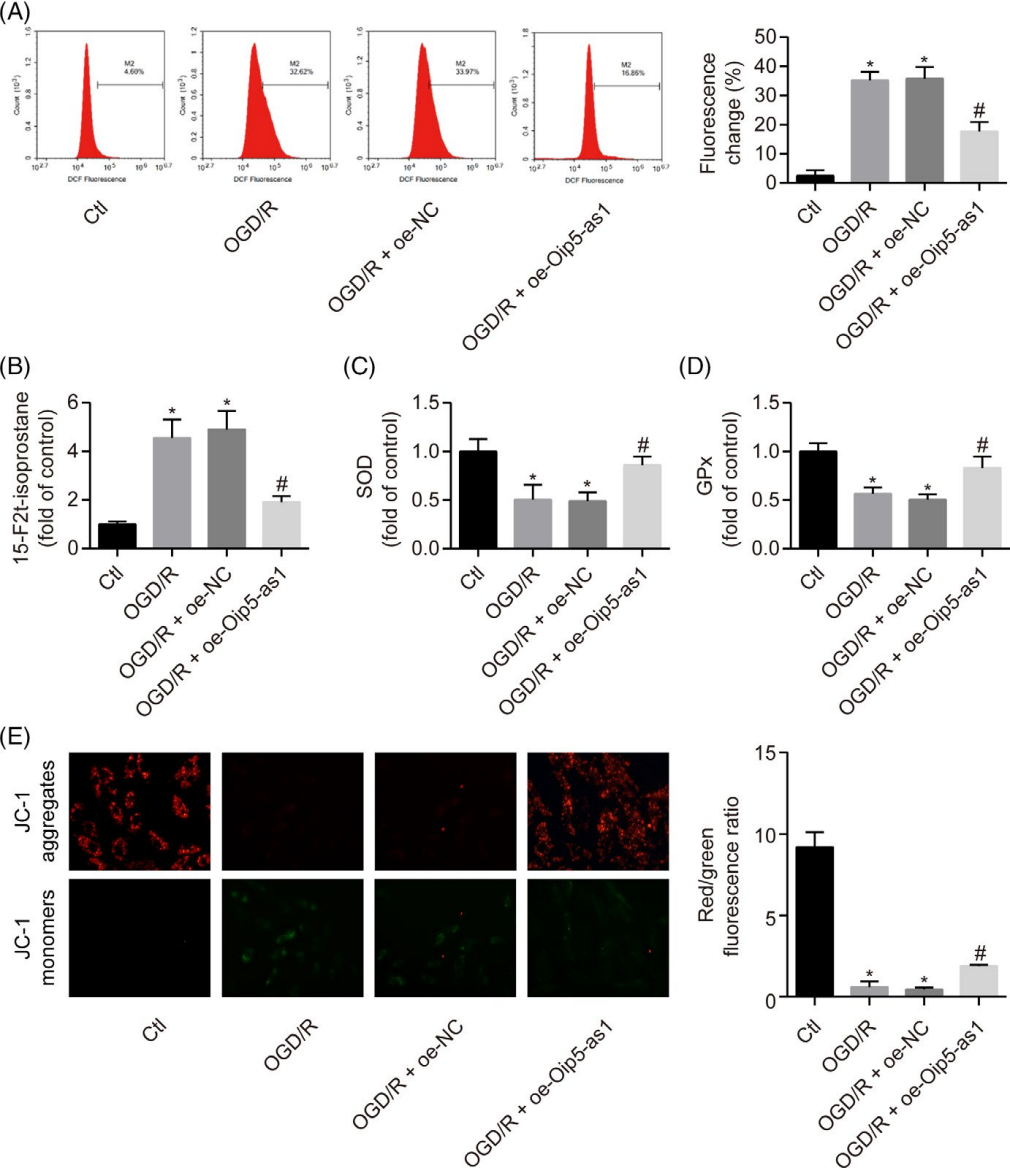
Oip5‐as1 attenuates OGD/R‐induced oxidative stress and mitochondrial membrane depolarization in H9c2 cells. A, Flow cytometric detection of ROS levels in H9c2 cells treated with Oip5‐as1 overexpression vector (oe‐Oip5‐as1) and OGD/R. B‐D, Measurement of oxidative stress markers (15‐F2t‐isoprostane, SOD and GPx) using the respective assay kits in H9c2 cells treated with oe‐Oip5‐as1 and OGD/R. E, Representative images and bar graphs of JC‐1 red/green cells treated with oe‐Oip5‐as1 and OGD/R. Data are expressed as mean ± SD (n = 3). *P < .05 vs the control group. ^#^ *P* < .05 vs the OGD/R + oe‐NC group

ROS‐triggered oxidative stress promotes apoptosis by directly targeting the mitochondria, which in turn, are the main endogenous sources of ROS. Flow cytometric analysis showed that Oip5‐as1 overexpression reduced the intracellular ROS levels in H9c2 cells under OGD/R conditions (Figure 3A). The oxidative damage parameters of H9c2 cells such as 15‐F2t‐isoprostane, GPx and SOD levels were measured using commercial kits. Oip5‐as1 overexpression significantly reduced the 15‐F2t‐isoprostane levels but increased SOD and GPx activities in OGD/R‐induced H9c2 cells (Figure 3B‐D).

JC‐1 staining was used to examine changes in MMP for the determination of mitochondrial membrane integrity. OGD/R exposure reduced the MMP in H9c2 cells, which was confirmed by increased green fluorescence and decreased red florescence of cells after JC‐1 staining. However, overexpression of Oip5‐as1 significantly restored the loss of MMP in OGD/R‐treated H9c2 cells (Figure 3E).

### 3.4 Oip5‐as1 functions as a miR‐29a ceRNA

**FIGURE 4:**
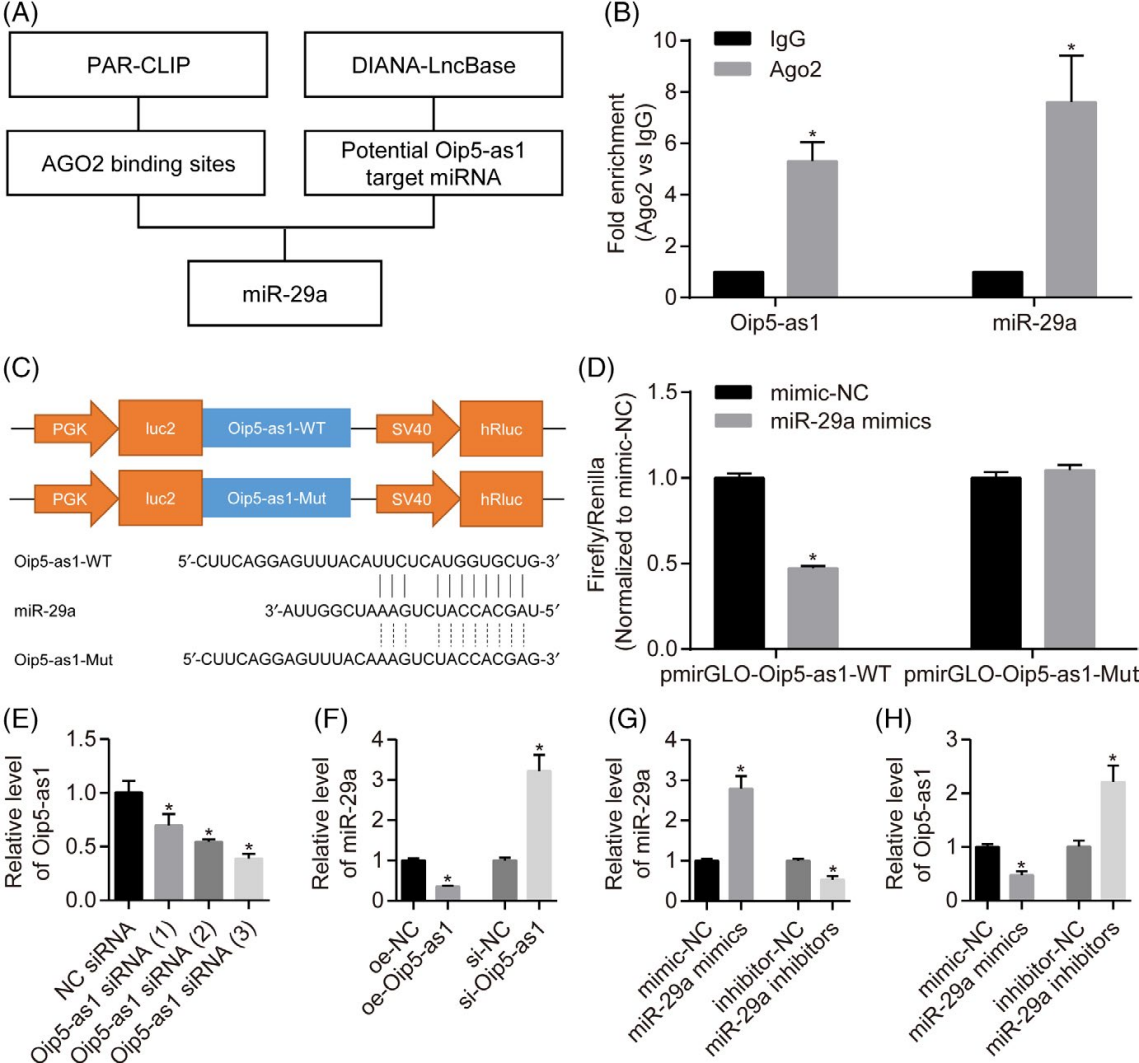
Oip5‐as1 functions as a ceRNA of miR‐29a. A, Prediction of Oip5‐as1 binding sites in miR‐29a and the Ago2 protein using the StarBase and DIANA‐LncBase databases. B, RT‐qPCR analysis of Oip5‐as1 and miR‐29a enrichment by Ago2 antibody and IgG antibody in RNA immunoprecipitation experiments. C, The sequences and positions of Oip5‐as1 containing the wild type and mutant binding sites of miR‐29a cloned into the pmirGLO luciferase reporter vectors. D, Luciferase activity in HEK293T cells co‐transfected with the reporter plasmid inserted with the wild‐type or mutated Oip5‐as1 sequences and miR‐29a mimics or mimic‐NC. E, Determination of knockdown efficiency for three siRNAs towards Oip5‐as1 (si‐Oip5‐as1) in H9c2 cells by RT‐qPCR. The Oip5‐as1 siRNA (3) was applied in all experiments. F, Relative level of miR‐29a expression in H9c2 cells transfected with the overexpression vector or siRNA of Oip5‐as1 and the corresponding negative control (NC). G, Relative levels of miR‐29a expression in H9c2 cells transfected with mimics or inhibitors and their corresponding NCs by RT‐qPCR analysis. H, Relative levels of Oip5‐as1 expression in H9c2 cells transfected with miR‐29a mimics or inhibitors. Data are expressed as mean ± SD (n = 3). *P < .05 vs the IgG, mimic‐NC, inhibitor‐NC or si‐NC groups

To understand the molecular mechanism by which Oip5‐as1 regulates MI/R injury, we analysed the miRNAs interacting with Oip5‐as1 using the DIANA‐LncBase^29^ and starBase^30^ tools. The results showed that Oip5‐as1 binds both miR‐29a and the Ago2 protein (Figure 4A). Ago2 protein is a key component of the RNA‐induced silencing complex (RISC). The anti‐Ago2 RIP assay was used to determine the role of endogenous Oip5‐as1 in miRNA‐containing ribonucleoprotein complexes. Oip5‐as1 and miR‐29a were preferentially enriched in Ago2‐containing beads compared to the control IgG (Figure 4B). These results suggested that Ago2 protein bound directly to Oip5‐as1 and miR‐29a.

Luciferase vectors of the wild type and mutant Oip5‐as1 were constructed to determine whether miR‐29a recognizes the predicted target site within Oip5‐as1. Dual‐luciferase assays (Figure 4C,D) showed that transfection with miR‐29a mimics significantly decreased the relative firefly luciferase activity of Oip5‐as1‐WT whereas the Oip5‐as1‐Mut luciferase activity was not affected.

To further study the relationship between Oip5‐as1 and miR‐29a, RT‐qPCR tests were performed to analyse the expression levels of Oip5‐as1 and miR‐29a in H9c2 cells. Three different sequences of Oip5‐as1 siRNAs were used to knockdown Oip5‐as1 expression in H9c2 cells. Transfection with Oip5‐as1 siRNA (3) exhibited the highest knockdown efficiency compared with other siRNAs (Figure 4E) and was thus chosen for subsequent experiments. In addition, Oip5‐as1 knockdown resulted in upregulation of miR‐29a expression whereas Oip5‐as1 overexpression downregulated the expression of miR‐29a in H9c2 cells (Figure 4F). When miR‐29a mimics and inhibitors were transfected into H9c2 cells (Figure 4G), the expression of Oip5‐as1 was negatively regulated (Figure 4H). These results indicate that Oip5‐as1 serves as a natural sponge for miR‐29a.

### 3.5 Inhibition of miR‐29a reduces oxidative stress injury under OGD/R conditions

**FIGURE 5:**
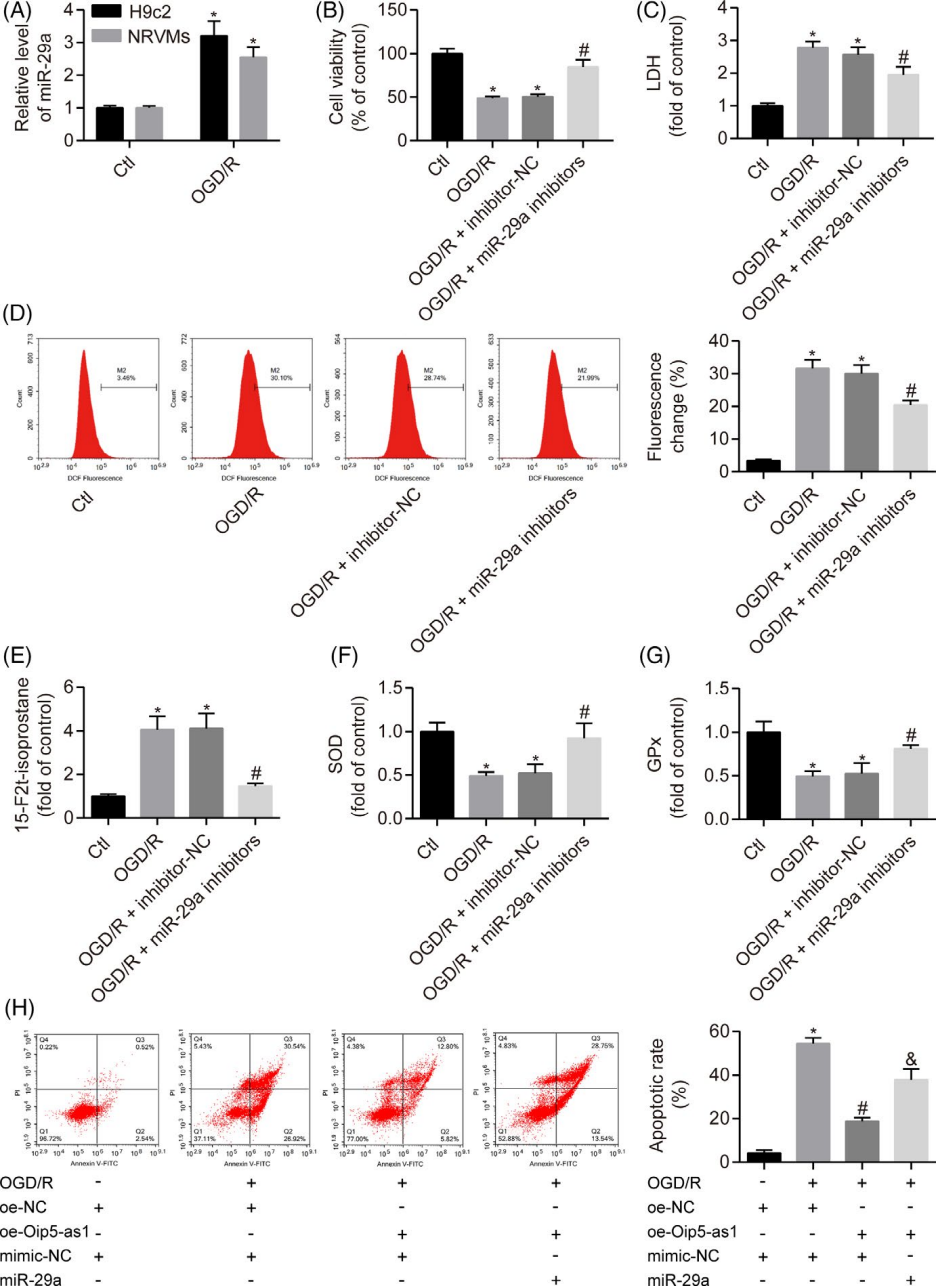
Inhibition of miR‐29a reduces oxidative stress injury under OGD/R conditions. A, Relative levels of miR‐29a expression in H9c2 cells and neonatal rat ventricular myocytes (NRVMs) treated with OGD/R. Cell viability (B) and LDH activity (C) of H9c2 cells transfected with miR‐29a inhibitors or negative control (inhibitor‐NC) under OGD/R conditions. D, Flow cytometric analysis of ROS levels in H9c2 cells treated with miR‐29a inhibitors and OGD/R. E‐G, Measurement of oxidative stress markers (15‐F2t‐isoprostane, SOD and GPx) using the respective assay kits in H9c2 cells treated with miR‐29a inhibitors and OGD/R. H, Cell apoptosis analysis by Annexin V/PI staining and flow cytometry in H9c2 cells treated with the Oip5‐as1 overexpression vector (oe‐Oip5‐as1) and miR‐29a mimics under OGD/R conditions. Data are expressed as mean ± SD (n = 3). *P < .05 vs the control or mimic‐NC + oe‐NC groups. ^#^ *P* < .05 vs the OGD/R + inhibitor‐NC or OGD/R + mimic‐NC + oe‐NC groups. ^&^ *P* < .05 vs the OGD/R + mimic‐NC + oe‐Oip5‐as1 group

We then evaluated the role of miR‐29a in oxidative stress injury under OGD/R conditions. miR‐29a was significantly upregulated in NRVMs, and H9c2 cells treated with OGD/R (Figure 5A). Inhibition of miR‐29a significantly reduced the cytotoxicity under OGD/R conditions as shown by increased cell viability and decreased LDH activity (Figure 5B,C). Moreover, miR‐29a inhibition decreased the levels of ROS and 15‐F2t‐isoprostane whereas it increased the activities of SOD and GPx in OGD/R‐treated H9c2 cells (Figure 5D‐G). Notably, miR‐29a overexpression prevented the anti‐apoptotic effect of oe‐Oip5‐as1 transfection in OGD/R‐treated H9c2 cells (Figure 5H).

### 3.6 miR‐29a‐mediated SIRT1 downregulation inactivates the AMPK/PGC1α pathway

**FIGURE 6:**
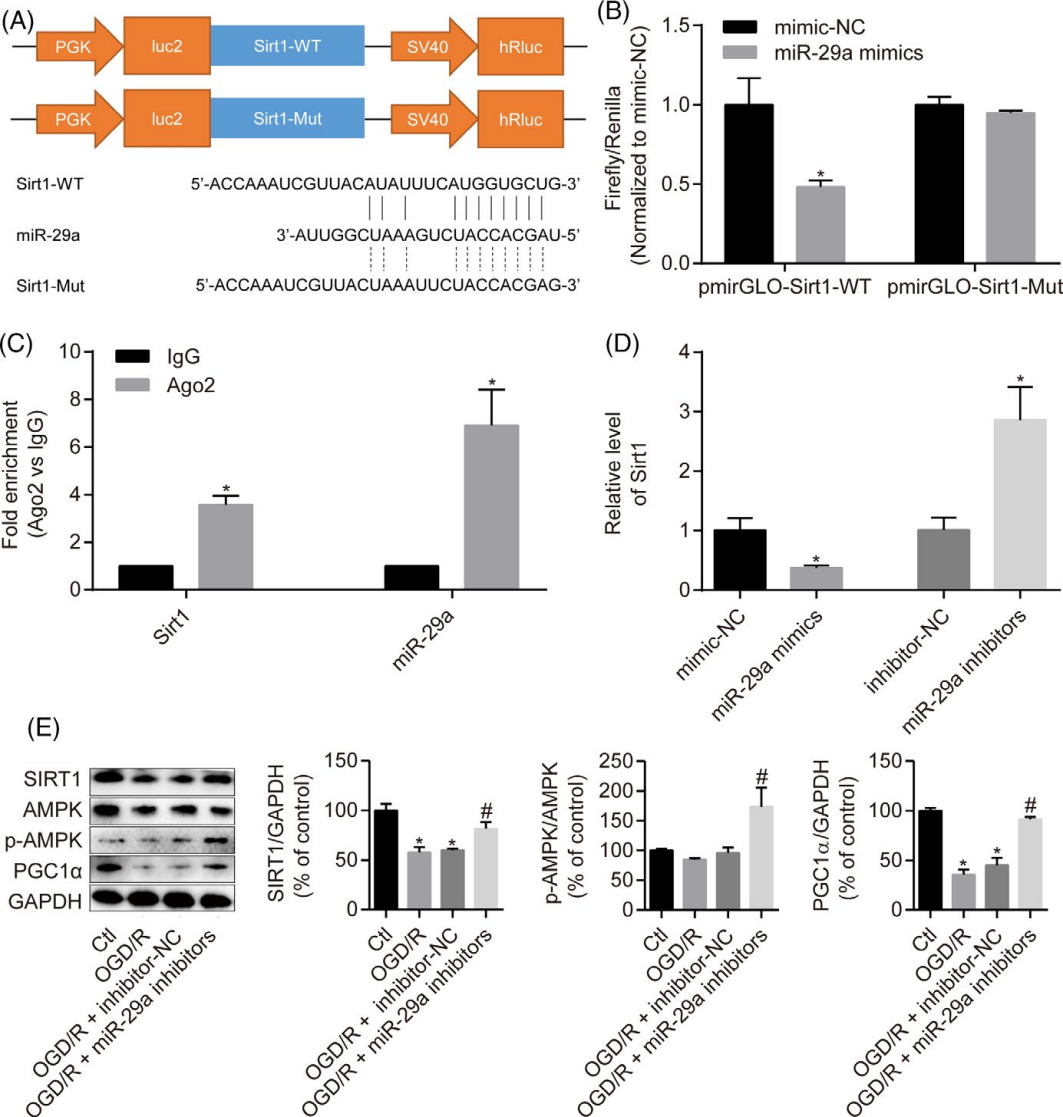
miR‐29a‐mediated SIRT1 downregulation inactivates the AMPK/PGC1α pathway. A, The sequences and positions of Sirt1 containing wild type and the mutant binding site of miR‐29a cloned into the pmirGLO luciferase reporter vector. B, Luciferase activity analysis in HEK293T cells co‐transfected with the reporter plasmid containing the wild‐type or mutated Sirt1 sequences and miR‐29a mimics or NC mimics. C, RT‐qPCR analysis of Oip5‐as1 and miR‐29a enrichment by the Ago2 antibody and IgG antibody in RNA immunoprecipitation experiments. D, Sirt1 expression in H9c2 cells transfected with miR‐29a mimics or inhibitors by RT‐qPCR. E, Western blot and densitometric analysis of SIRT1, p‐AMPK and PGC1α levels in H9c2 cells treated with miR‐29a inhibitors and OGD/R. Data are expressed as mean ± SD (n = 3). *P < .05 vs the mimic‐NC, inhibitor‐NC, IgG or control groups. ^#^ *P* < .05 vs the OGD/R + inhibitor‐NC group

Bioinformatics analysis was performed using the TargetScan tool^31^ to explore the downstream signalling mechanisms for the role of miR‐29a in H9c2 cells. The results showed a miR‐29a binding site at the 3′‐UTR of SIRT1 mRNA (Figure 6A). Based on these results, luciferase gene reporter analysis and RIP assay were performed to help understand the interaction of miR‐29a and Sirt1. We generated the recombinant pmirGLO reporter plasmids expressing Sirt1‐WT or Sirt1‐Mut and found that co‐transfection with miR‐29a mimics significantly reduced luciferase activity of Sirt1‐WT. However, there was no significant difference within the Sirt1‐Mut groups transfected with either the miR‐29a mimics or mimic‐NC (Figure 6B). Moreover, Sirt1 and miR‐29a expression was significantly increased in Ago2‐RIP but not in IgG‐RIP (Figure 6C).

We then evaluated the regulatory effect of miR‐29a on Sirt1. RT‐qPCR analysis showed that transfection of H9c2 cells with miR‐29a mimics led to lower levels of Sirt1, whereas transfection of cells with miR‐29a inhibitors resulted in higher Sirt1 levels (Figure 6D).

The SIRT1/AMPK/PGC1α signalling pathway is known to be associated with MI/R injury. Western blot analysis results revealed that inhibition of miR‐29a upregulated SIRT1 expression and induced AMPK phosphorylation and PGC1α expression in OGD/R‐treated H9c2 cells (Figure 6E). Collectively, miR‐29a exacerbates H9c2 cell injury induced by OGD/R through inhibition of the SIRT1/AMPK/PGC1α pathway.

### 3.7 Oip5‐as1 upregulates SIRT1 to activate the AMPK/PGC1α pathway

**FIGURE 7:**
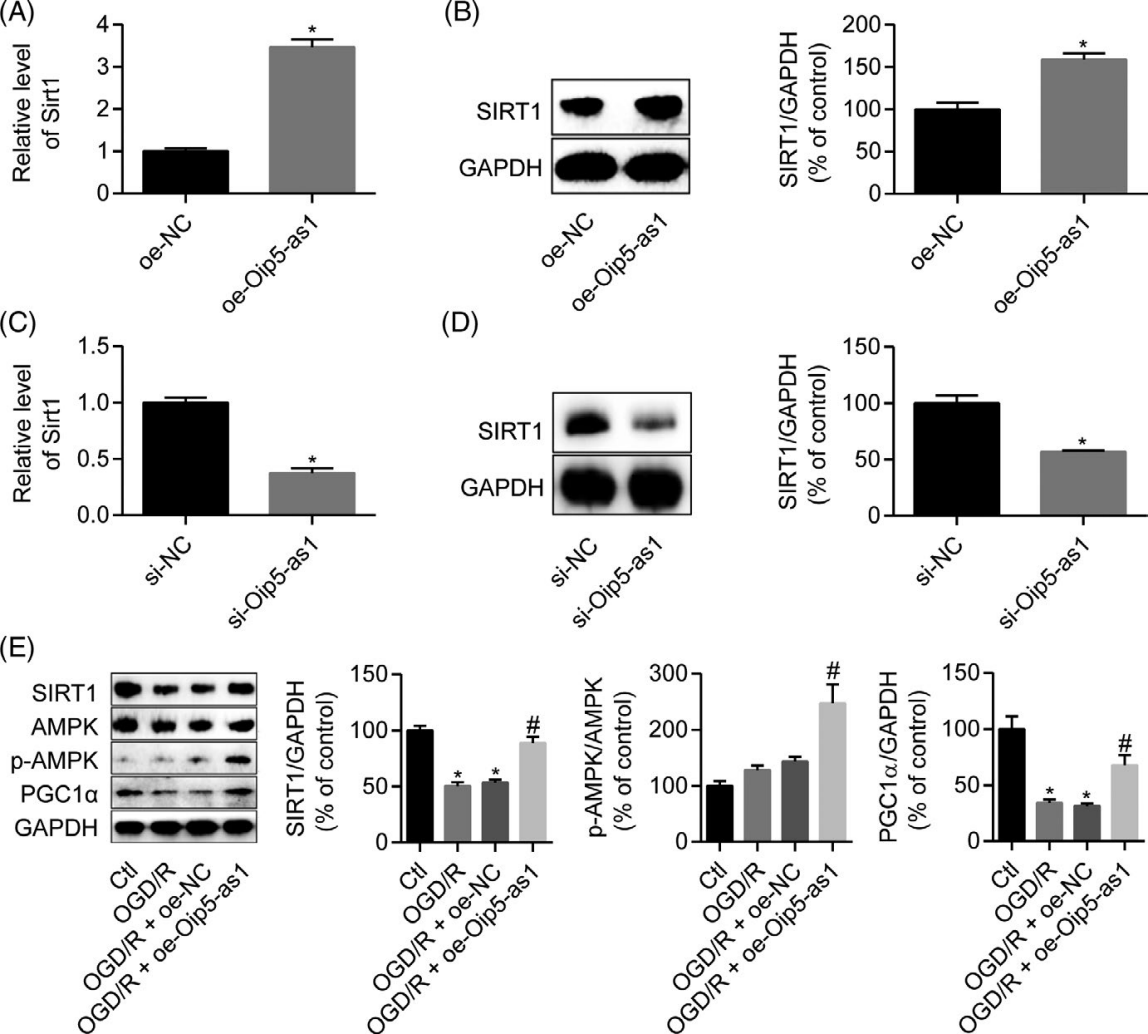
Oip5‐as1 upregulates SIRT1 leading to activation of the AMPK/PGC1α pathway. A, RT‐qPCR analysis of Sirt1 expression in H9c2 cells transfected with the Oip5‐as1 overexpression vector (oe‐Oip5‐as1). B, Western blot and densitometric analysis of SIRT1 in H9c2 cells transfected with oe‐Oip5‐as1. C, RT‐qPCR analysis of Sirt1 expression in H9c2 cells transfected with the siRNA targeting Oip5‐as1 (si‐Oip5‐as1). D, Western blot and densitometric analysis of SIRT1 in H9c2 cells transfected with si‐Oip5‐as1. E, Western blot and densitometric analysis of SIRT1, p‐AMPK and PGC1α levels in H9c2 cells treated with oe‐Oip5‐as1 and OGD/R. Data are expressed as mean ± SD (n = 3). *P < .05 vs the oe‐NC, si‐NC or control groups. ^#^ *P* < .05 vs the OGD/R + oe‐NC group

Because Oip5‐as1 had been found to interact with miR‐29a, we further explored whether Oip5‐as1 could regulate SIRT1, which is the downstream target of miR‐29a. Overexpression of Oip5‐as1 upregulated SIRT1 expression at the mRNA (Figure 7A) and protein (Figure 7B) levels in H9c2 cells, whereas Oip5‐as1 knockdown by siRNAs significantly reduced SIRT1 expression at the mRNA (Figure 7C) and protein (Figure 7D) levels. Western blot analysis further showed that transfection of H9c2 cells with oe‐OIP5‐as1 activated the SIRT1/AMPK/PGC1α pathway that was previously inhibited by OGD/R treatment (Figure 7E).

### 3.8 Oip5‐as1 protects H9c2 cells against OGD/R injury by targeting miR‐29a and the SIRT1/AMPK/PGC1α pathway

**FIGURE 8:**
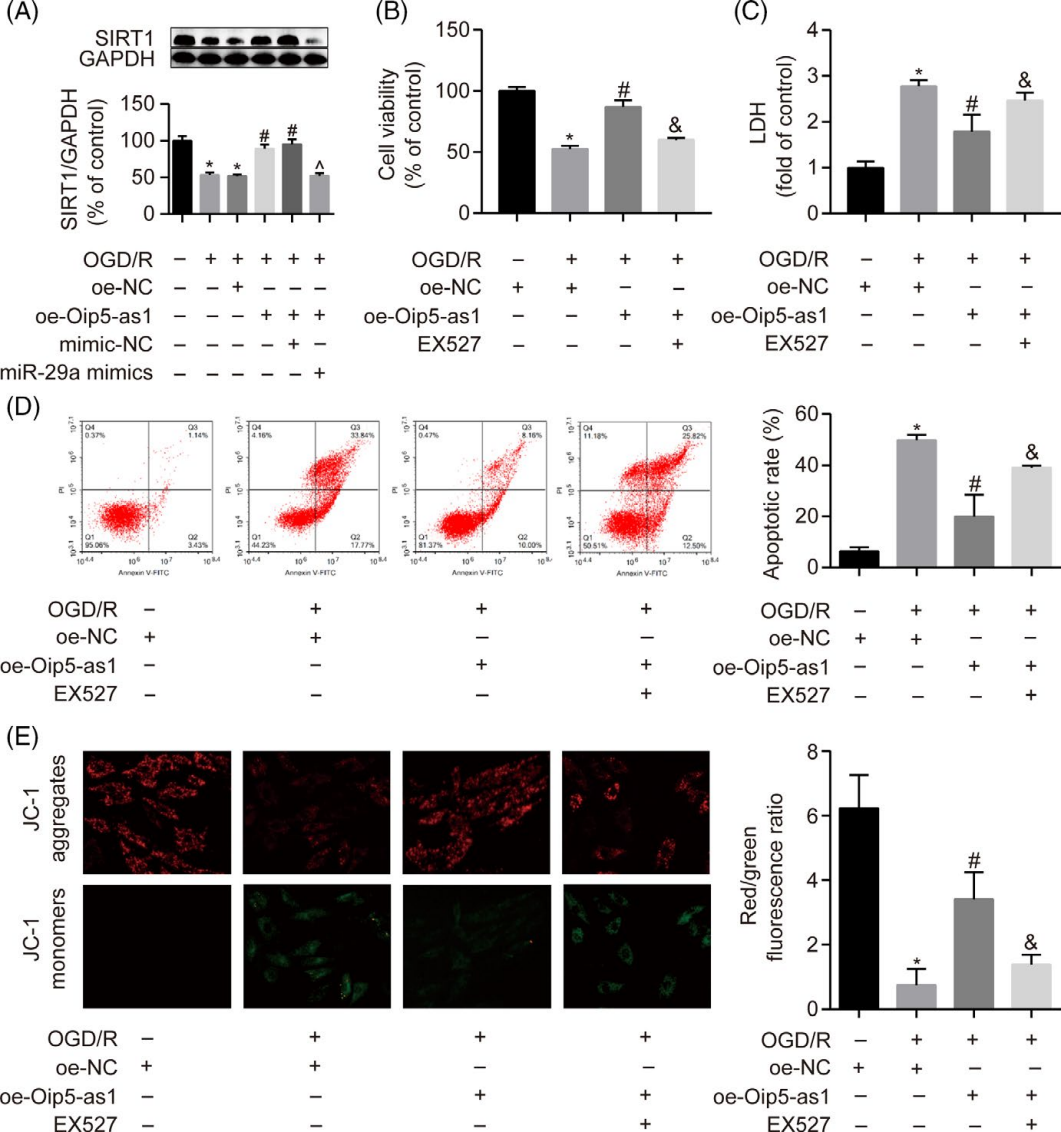
Oip5‐as1 protects H9c2 cells from OGD/R injury through the miR‐29a/SIRT1/AMPK/PGC1α pathway. A, Western blot and densitometric analysis of SIRT1 expression in H9c2 cells transfected with the Oip5‐as1 overexpression vector (oe‐Oip5‐as1) and miR‐29a mimics. Cell viability (B) and LDH activity (C) of H9c2 cells treated with oe‐Oip5‐as1 and EX527 (SIRT1 inhibitor) under OGD/R conditions. D, Cell apoptosis analysis by Annexin V/PI staining and flow cytometry in H9c2 cells treated with oe‐Oip5‐as1 and EX527 under OGD/R conditions. E, Representative images and bar graphs of JC‐1 red/green cells treated with oe‐Oip5‐as1 and EX527 under OGD/R conditions. Data are expressed as mean ± SD (n = 3). *P < .05 vs the oe‐NC or control groups. ^#^ *P* < .05 vs the OGD/R + oe‐NC group. ^&^ *P* < .05 vs the OGD/R + oe‐Oip5‐as1 group. ^^^ *P* < .05 vs the OGD/R + oe‐Oip5‐as1 + mimic‐NC group

To confirm whether miR‐29a and its target SIRT1 mediated the effects of Oip5‐as1, rescue experiments were performed by transfecting H9c2 cells with miR‐29a mimics or by treating them with EX527 (SIRT1 inhibitor). Upregulation of SIRT1 expression by transfection with oe‐OIP5‐as1 was inhibited by miR‐29a mimics in OGD/R‐induced H9c2 cells (Figure 8A). Overexpression of Oip5‐as1 significantly improved cytotoxicity, apoptosis and MMP in OGD/R‐treated H9c2 cells. However, the beneficial effects of oe‐Oip5‐as1 transfection were significantly reversed by treatment with EX527 (Figure 8B‐E). These results further confirmed that overexpression of Oip5‐as1 activated the SIRT1/AMPK/PGC1α pathway by sponging miR‐29a to protect H9c2 cells against OGD/R injury.

### 3.9 Oip5‐as1 upregulation ameliorates MI/R injury in rats

**FIGURE 9:**
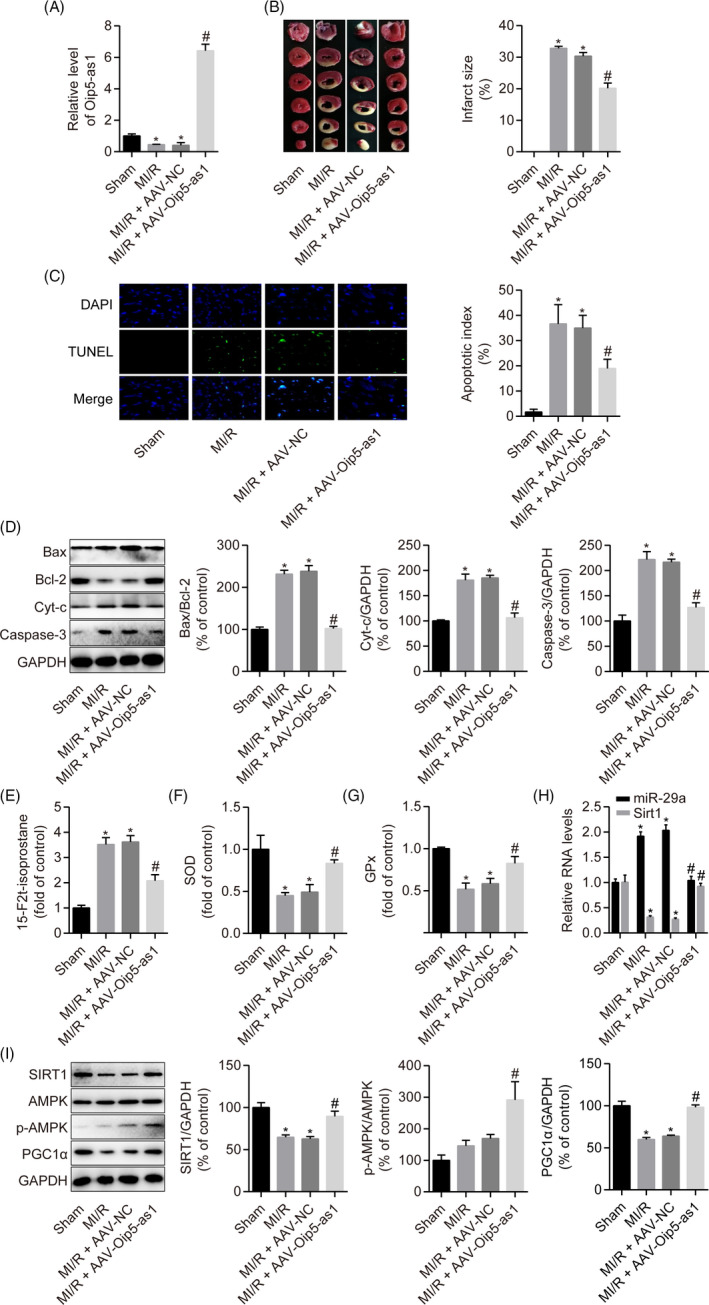
Oip5‐as1 upregulation ameliorates MI/R injury in rats. A, RT‐qPCR analysis of the relative Oip5‐as1 expression levels in rat hearts. B, Representative images of heart sections stained with TTC and the quantitative analysis of myocardial infarct size. C, Representative pictures of cardiomyocyte apoptosis determined by the TUNEL assay and corresponding quantitative analysis. D, Western blot and densitometric analysis of the expression of apoptosis marker proteins (Bax, Bcl‐2, Cyt‐c and Caspase‐3) in rat hearts. E‐G, Measurement of oxidative stress markers (15‐F2t‐isoprostane, SOD and GPx) using the respective assay kits in rat hearts. H, RT‐qPCR analysis of the relative expression levels of miR‐29a and Sirt1 in rat hearts. I, Western blot and densitometric analysis of SIRT1, p‐AMPK and PGC1α levels in heart samples from rats. Data are expressed as mean ± SD (n = 6). *P < .05 vs the sham group. ^#^ *P* < .05 vs the MI/R + AAV‐NC group

To further confirm the role of Oip5‐as1 in MI/R injury *in vivo*, we established a rat model of MI/R injury after AAV‐Oip5‐as1 injection. RT‐qPCR revealed that cardiac tissues from the MI/R group showed relatively lower expression levels of Oip5‐as1 compared to the sham group. AAV‐Oip5‐as1 injection effectively increased Oip5‐as1 expression by 6.4‐fold in the hearts of MI/R‐injured rats (Figure 9A). TTC staining showed that Oip5‐as1 upregulation significantly inhibited the MI/R‐triggered increase of the infarct size in cardiac tissues (Figure 9B). As shown in Figure 9C,D, MI/R stimulation significantly increased the proportion of apoptotic cells and Bax/Bcl‐2 ratio, Cyt‐c and Caspase‐3 levels, whereas Oip5‐as1 upregulation relieved these MI/R‐triggered effects. Upregulation of Oip5‐as1 significantly reversed an increase in 15‐F2t‐isoprostane levels and a decrease in the SOD and GPx activities induced by MI/R (Figure 9E‐G).

Additionally, Oip5‐as1 overexpression significantly decreased the level of miR‐29a and increased Sirt1 expression under MI/R conditions in rats (Figure 9H). Western blot analysis showed that Oip5‐as1 overexpression promoted the expression levels of SIRT1, AMPK phosphorylation and PGC1α in MI/R‐injured rats (Figure 9I). These results indicate that Oip5‐as1 plays an important role in cardiomyocyte apoptosis in MI/R rats.

## 4 DISCUSSION

In this study, we demonstrated that Oip5‐as1 is downregulated after MI/R injury. Gain‐of‐function analyses using the CRISPR/dCas9 SAM system or AAV delivery indicated that following MI/R injury, Oip5‐as1 prevents apoptosis by acting as a ceRNA for miR‐29a. We identified SIRT1 as the target of miR‐29a that regulates ROS‐induced mitochondrial damage and apoptosis in H9c2 cells.

Studies have demonstrated that lncRNA regulates the expression of protein‐coding genes involved in cell development, differentiation, metabolic regulation, signal transduction, proliferation and apoptosis.^8^, ^10^, ^11^ The first lncRNA identified as a marker of AMI was the MIAT1 (myocardial infarction‐associated transcript 1).^32^ Downregulation of MIAT1 alleviates oxidative stress and myocardial apoptosis after MI/R injury by inhibiting NF‐κB signalling.^33^ Overexpression of lncRNA H19 attenuates MI/R injury in mice and H_2_O_2_‐induced cardiomyocyte injury by suppressing miR‐877‐3p/Bcl‐2‐mediated mitochondrial apoptosis.^21^ Therefore, investigating the underlying mechanisms of lncRNAs will help develop novel strategies for treating MI/R injury.^10^, ^11^ OIP5‐AS1 is a novel lncRNA reported to participate in the development of malignant tumours.^18^ OIP5‐AS1 is upregulated and acts as an oncogene in multiple malignancies such as breast cancer,^14^ cervical cancer,^15^ glioma^16^, ^17^ and hemangioma.^34^ Recently, OIP5‐AS1 was found to be highly expressed in a human umbilical vein endothelial cell (HUVEC) model of atherosclerosis.^35^ OIP5‐AS1 promotes apoptosis and inhibits proliferation in HUVECs treated with oxidative low‐density lipoprotein (ox‐LDL).^35^ Yang et al^36^ similarly reported that OIP5‐AS1 promotes atherosclerosis in human vascular smooth muscle cells treated with ox‐LDL. Results from these two previous studies^35^, ^36^ implicate OIP5‐AS1 in atherosclerosis development. However, little was known about the role of OIP5‐AS1 in myocardial diseases. In this study, we found that Oip5‐as1 was downregulated in NRVMs and H9c2 cells upon OGD/R exposure and in MI/R‐injured rat hearts. Notably, Oip5‐as1 overexpression reduced oxidative stress, improved mitochondrial function and inhibited apoptosis. Our findings thus suggest that Oip5‐as1 plays a cardioprotective role during MI/R.

Similar to other lncRNAs, Oip5‐as1 acts as a ceRNA by sponging miRNAs and promotes the expression of miRNA targets. A study by Zeng et al^14^ showed that OIP5‐AS1 acts as a ceRNA by sequestering miR‐129‐5p, which hindered binding to its target SOX2. Liu et al^16^ demonstrated that OIP5‐AS1 functions as a ceRNA of miR‐367‐3p to regulate CEBPA expression. In this study, we found that Oip5‐as1 contains a binding site for miR‐29a and inhibits the function of miR‐29a through a base complementation mechanism. Previous studies^37^, ^38^ reported that miR‐29a is upregulated after MI/R injury and that inhibition of miR‐29a protected H9c2 cells against OGD/R‐induced apoptosis. Another recent study^39^ showed that downregulation of miR‐29a protects MI/R injury and inhibits oxidative stress and pyroptosis. In line with previous findings, our results indicate that miR‐29a promotes cardiomyocyte apoptosis and MI/R injury. Moreover, we show that miR‐29a binds to the 3′‐UTR of Sirt1. Upregulation of miR‐29a reduced the expression level of SIRT1 in H9c2 cells.

Gain‐ and loss‐of‐function experiments have previously demonstrated the role of SIRT1 as a critical signal mediating MI/R injury. Hsu et al^19^ reported that transgenic mice with cardiac‐specific Sirt1 overexpression exhibited decreased oxidative stress and apoptosis in response to MI/R. Wang et al^20^ reported that mice with cardiomyocyte‐specific SIRT1 knockout showed increased susceptibility to MI/R injury as evidenced by increased infarct size and impaired cardiac function. Further mechanistic experiments showed that SIRT1 activated LKB1 (liver kinase B1) leading to the increased AMPK phosphorylation. Activation of AMPK maintains cellular energy metabolism and promotes cell survival under ischaemic and oxidative stress conditions.^20^, ^40^ AMPK confers cardioprotection by directly phosphorylating its target proteins or via transcriptional control of target genes such as PGC1α, FoxO3 (forkhead box O3) and p300.^20^, ^40^ PGC1α regulates energy homoeostasis, oxidative metabolism and cardiac mitochondrial function.^41^ High PGC1α expression accelerates mitochondrial function recovery and improves cell survival during MI/R injury.^42^ Activation of the SIRT1/AMPK/PGC1α signalling pathway is known to mediate the cardioprotective effects of multiple drugs such as rutin,^24^ melatonin,^43^ icariin^44^ and fibroblast growth factor 21.^45^ In this study, we found that the highly selective SIRT1 inhibitor EX527 prevents AMPK phosphorylation and PGC1α expression induced by Oip5‐as1 overexpression. Additionally, miR‐29a overexpression reversed the effect of Oip5‐as1 on SIRT1 expression. Our results suggest that Oip5‐as1 targets miR‐29a and regulates its downstream effect on the SIRT1/AMPK/PGC1α pathway.

Apoptosis is initiated by two major pathways, the intrinsic pathway (mitochondria‐ and endoplasmic reticulum‐mediated) and the extrinsic pathway (death receptor‐mediated).^7^ The mitochondrial apoptosis pathway has been confirmed to be involved in MI/R injury.^5^ Mitochondria are the major sources of ROS under MI/R conditions. High ROS levels can damage biological macromolecules and the mitochondria, thereby initiating a vicious cycle leading to the exacerbation of ROS formation and mitochondrial dysfunction.^7^ Loss of MMP indicates disruption of mitochondrial membrane function, which triggers the release of Cyt‐c from the mitochondria to the cytoplasm.^7^ Bax, a pro‐apoptotic protein, increases mitochondrial permeability and Cyt‐c release, leading to Caspase‐3 activation and apoptosis.^7^ Bcl‐2 neutralizes the pro‐apoptotic activity of Bax by preventing conformational changes or translocation of Bax to the mitochondria.^7^ Thus, strategies that improve mitochondrial function can be exploited to restore cardiac function after MI/R injury.^5^ The effect of Oip5‐as1 on apoptosis in MI/R injury via the mitochondria‐dependent pathway remains unclear. The present study demonstrates that Oip5‐as1 decreases excessive ROS production, maintains MMP and prevents MI/R‐induced apoptosis. We also found that Oip5‐as1, a ceRNA of miR‐29a, reduced the release of Cyt‐c from the mitochondria to the cytoplasm and inhibited Caspase‐3 activation. Activation of the miR‐29a target Sirt1 alleviated oxidative stress‐triggered ROS accumulation and the related oxidative damage. Furthermore, treatment with EX527 reversed the protective effects of Oip5‐as1 on mitochondrial function, oxidative stress and apoptosis in MI/R injury. Thus, our study reports an important role of the Oip5‐as1/miR‐29a/SIRT1/AMPK/PGC1α signalling axis in MI/R injury.

## 5 CONCLUSIONS

**FIGURE 10:**
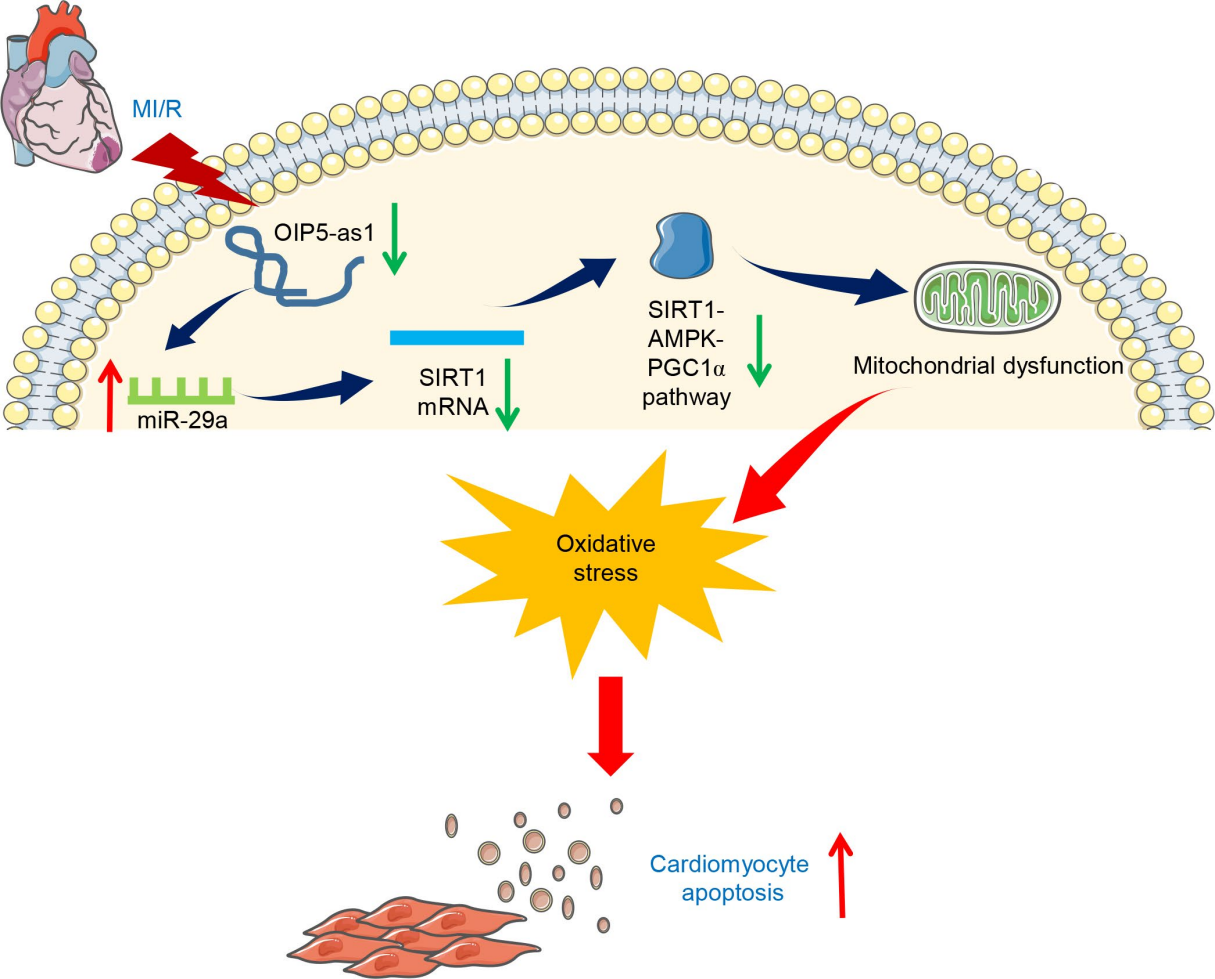
Schematic representation of the proposed mechanism of Oip5‐as1 involvement in MI/R injury. Oip5‐as1 acts as a ceRNA to sponge miR‐29a and upregulates SIRT1 expression and SIRT1 downstream effectors, thereby attenuating cardiomyocyte apoptosis

For the first time, we demonstrate that Oip5‐as1 suppresses miR‐29a leading to activation of the SIRT1/AMPK/PGC1α pathway, which attenuates mitochondrial dysfunction, oxidative stress and apoptosis during MI/R injury (Figure 10). Our findings offer a new therapeutic target for developing drugs against MI/R injury.

## CONFLICT OF INTEREST

The authors declare that there are no competing interests associated with the manuscript.

## AUTHOR CONTRIBUTIONS

NX and ZZ conceived and designed the study. NX, PS, LC, XJ, WJ, SS and YY performed the study. NX, PS, LC, XJ and WJ analysed the data. NX and ZZ wrote the manuscript. PS, LC, XJ, WJ, SS and YY critically revised intellectual content of the manuscript. The authors sincerely thank Yingdong Li from Traditional Chinese Medicine of Gansu Province for their support during the study.

## Supporting information

Table S1‐S3

## Data Availability

All data sets for this study are included in the manuscript.
