# Supplementary material for: lncRNA Oip5‐as1 attenuates myocardial ischaemia/reperfusion injury by sponging miR‐29a to activate the SIRT1/AMPK/PGC1α pathway
Source: Cell Prolif. 2020 May 28;53(6):e12818. doi: 10.1111/cpr.12818 (PMC7309946; doi:10.1111/cpr.12818)
Supplement: Supplementary file 1 — Table S1‐S3 [file CPR-53-e12818-s001.docx]

**Supplementary Table 1.** siRNA sequence used in this study

| siRNA | Sense sequence (5′ to 3′) | Anti-sense sequence (5′ to 3′) |  |
| --- | --- | --- | --- |
| Oip5-as1 siRNA (1) | GGACUUGUCUGAUUAGGUUTT | AACCUAAUCAGACAAGUCCTT | |
| Oip5-as1 siRNA (2) | CCUUUGGACUUACAGGAAUTT | AUUCCUGUAAGUCCAAAGGTT |  |
| Oip5-as1 siRNA (3) | CCUGGUAUGUGGCACAUUUTT | AAAUGUGCCACAUACCAGGTT |  |
| NC siRNA | UUCUCCGAACGUGUCACGUTT | ACGUGACACGUUCGGAGAATT |  |

**Supplementary Table 2.** Primer sequence used in this study

| Gene | Forward primer (5′ to 3′) | Reverse primer (5′ to 3′) |
| --- | --- | --- |
| Oip5-as1 | GTGTTGTGGAGATTGAGGCAGGAG | GGCAAGGTGAAGGACAGACAGC |
| Sirt1 | GCTCGCCTTGCTGTGGACTTC | GTGACACAGAGATGGCTGGAACTG |
| β-actin | TGTCACCAACTGGGACGATA | GGGGTGTTGAAGGTCTCAAA |
| U6 | TGGAACGCTTCACGAATTTGCG | GGAACGATACAGAGAAGATTAGC |

**Supplementary Table 3.** Information on antibodies used for western blot

| Antibody | Catalog number | Dilution ratio | Company |
| --- | --- | --- | --- |
| AMPKα (D5A2) Rabbit mAb | 5831 | 1:1000 | Cell Signaling Technology |
| Anti-Bax Antibody | ab32503 | 1:10000 | Abcam |
| Bcl-2 Antibody | MAB8272 | 1:10000 | R&D Systems |
| Cleaved Caspase-3 (Asp175) (5A1) Rabbit mAb | 9664 | 1:1000 | Cell Signaling Technology |
| Cytochrome c | 10993-1-AP | 1:1000 | Proteintech Group |
| Phospho-AMPKα (Thr172) (40H9) Rabbit mAb | 2535 | 1:1000 | Cell Signaling Technology |
| SirT1 (D1D7) Rabbit mAb | 9475 | 1:1000 | Cell Signaling Technology |
| GAPDH Antibody | 10494-1-AP | 1:10000 | Proteintech Group |
